# Domestic violence against women in eastern India: a population-based study on prevalence and related issues

**DOI:** 10.1186/1471-2458-9-129

**Published:** 2009-05-09

**Authors:** Bontha V Babu, Shantanu K Kar

**Affiliations:** 1 Regional Medical Research Centre, Indian Council of Medical Research, Bhubaneswar-751 023, Orissa, India; 2 Indian Council of Medical Research, New Delhi-110 029, India

## Abstract

**Background:**

Violence against women is now widely recognised as an important public health problem, owing to its health consequences. Violence against women among many Indian communities on a regularly basis goes unreported. The objective of this study is to report the prevalence and other related issues of various forms of domestic violence against women from the eastern zone of India.

**Methods:**

It is a population-based study covering both married women (n = 1718) and men (n = 1715) from three of the four states of Eastern India selected through a systematic multistage sampling strategy. Interviews were conducted using separate pre-piloted structured questionnaires for women (victimization) and men (perpetration). Women were asked whether their husband or any other family members committed violent acts against them. And men were asked whether they had ever perpetrated violent acts against their wives. Three principle domestic violence outcome variables (physical, psychological and sexual violence) were determined by response to a set of questions for each variable. In addition, data on socio-economic characteristics were collected. Descriptive statistics, bi- and multivariate analyses were done.

**Results:**

The overall prevalence of physical, psychological, sexual and any form of violence among women of Eastern India were 16%, 52%, 25% and 56% respectively. These rates reported by men were 22%, 59%, 17% and 59.5% respectively. Men reported higher prevalence of all forms of violence apart from sexual violence. Husbands were mostly responsible for violence in majority of cases and some women reported the involvement of husbands' parents. It is found that various acts of violence were continuing among majority of women who reported violence. Some socio-economic characteristics of women have significant association with the occurrence of domestic violence. Urban residence, older age, lower education and lower family income are associated with occurrence of domestic violence. Multivariate logistic regressions revealed that the physical violence has significant association with state, residence (rural or urban), age and occupation of women, and monthly family income. Similar associations are found for psychological violence (with residence, age, education and occupation of the women and monthly family income) and sexual violence (with residence, age and educational level of women).

**Conclusion:**

The prevalence of domestic violence in Eastern India is relatively high compared to majority of information available from India and confirms that domestic violence is a universal phenomenon. The primary healthcare institutions in India should institutionalise the routine screening and treatment for violence related injuries and trauma. Also, these results provide vital information to assess the situation to develop public health interventions, and to sensitise the concerned agencies to implement the laws related to violence against women.

## Background

Violence against women is widely recognised as an important public health problem, owing to its substantial consequences for women's physical, mental and reproductive health [1-5]. This recognition was strengthened globally by resolutions of various international fora including fourth World Conference on Women in 1995 in Beijing [6]. In India, the problem has been highlighted after legislation against domestic violence in 2005, popularly known as the Protection of Women from Domestic violence Act [7]. Research across the world has provided increasing evidence of the problem of violence against women [8,9].

India possessed several communities which are distinct in their geography, language and culture. In several places of India, violence faced by women on a regularly basis goes unreported even in newspapers, where as newspapers often carry reports about young women being burnt alive or dying due to unnatural causes in unnatural circumstances [10]. Estimates of prevalence of domestic violence within India vary widely (from 18% to 70%, with differences in study methodology) [10-20], and it is realized that the magnitude of the problem has not been accounted well from several parts of India. There are very few studies covering the population across the country [14-16,18]. The third national family health survey revealed that there is considerable variation across the states in the prevalence of domestic violence [18]. A closer scrutiny of the prevalence rates reveals that domestic violence is a country-wide phenomenon with some variations between states, as these states differ from each other in overall socio-economic development and women's status [18,21]. A few community-based micro-studies are available from northern [11,19], southern [11,17] and western states [10] of India. However, community-based studies are not available from eastern part of India. Also, the available community-based studies are limited to physical violence. The third national family health survey revealed that more than a third of women in India have been physically mistreated by their husbands or other family members [18]. Some community-based surveys suggested that physical violence has been experienced by 21 to 48% of women in different settings in India [10,11,15,20]. The above estimates are corroborated by studies investigating reporting patterns of men. And 21 to 40% of men in different studies reported perpetrating physical violence [12,13,19,20]. Evidence on psychological violence is limited. Available community-based studies suggested that psychological violence ranged from 23% to 72% [10,11,13,15]. Evidence on sexual violence, as in the case of psychological violence, is also limited. A multi-site study revealed that 15% of sampled women reported one or more incidents of forced sex [15]. A study carried out in a district in Western India reported that 20% of the women reporting physical violence described abuse of sexual nature [10]. Studies with men revealed that 9% [12] to 26% [19] and 50% [13] of men reported perpetration of sexual violence. It is worth-noting that majority of the studies from India are based on the investigations on married women. A few studies are based on reporting of men [12,13,19,20]. In addition to above prevalence studies, there are a few qualitative studies to support the extent of burden of domestic violence in India [22-24].

We hypothesize that domestic violence is wide-spread phenomenon and variation in its prevalence occur across the eastern Indian states, as these states differ from each other in overall development. Also, it is hypothesized that differences occur within the population of these states based on some socio-economic characteristics such as habitation (rural or urban residence), age, religion/caste affiliation, education, occupation and income. The purpose of the present study is to report the prevalence of various forms of domestic violence against women and to examine various related issues from the eastern zone of India. The term domestic violence is usually taken to mean partner abuse, specifically violence perpetrated by male partner. However, it may also be used to refers to violence perpetrated by any member of the household towards the women [25]. However, this paper deals with the violence faced by women, perpetrated by their husbands and other family members within their conjugal homes.

## Methods

### Study area and participants

The eastern zone of India possessed four states namely, Orissa, West Bengal, Bihar and Jharkhand. Of these four states, three states (Orissa, West Bengal and Jharkhand) were selected to have a wider representation of the zone. The population of these states was 31.7 million, 80.2 million and 26.9 million in the year 2001 [26]. This study was a cross-sectional study. The participants were both men and women. The study involved collecting quantitative data through structured questionnaires. The questionnaire for women included items on socio-economic details and domestic violence experience. To assess domestic violence exposure, women were asked several questions on various behaviours of violence (see Annexure 1a in Additional file 1). Questions were posed to get their experience to a specific act of violence during their life time as well as during last twelve months. These behaviours and corresponding questions have been identified to constitute domestic violence based on previous studies in other settings [1,27,28]. The questionnaire for men included similar questions about his perpetration of violence against his wife (see Annexure 1b in Additional file 1). A multiphase process was used to develop these questionnaires to ensure that it was culturally and linguistically appropriate. These questionnaires were prepared initially in English and translated into the languages of the study states (Oriya in Orissa, Bengali in West Bengal and Hindi in Jharkhand). The questions, which were in above languages were back translated to English, by those who are not involved in this study to ensure semantic and content validity. The translated questionnaires were further reviewed for linguistic reliability and correctness by the study staff. Later the questionnaires were piloted to check appropriateness, clarity and flow of questions among some respondents, but from the villages that were not included in the study. In addition, piloting provided practice to the research staff, who collected data using these questionnaires.

All the interviews were held in local language of the state. Interviews took place in a private place in or outside the respondents' home, and care has been taken to avoid presence of other family/community members during interviews. If some one comes nearer during interview, the discussion on general health was made and the interview was restarted after the third person has retired. Interviewers stressed that honest responses were needed during interview to gain insight into the issue. Participants were assured of the confidentiality of their responses. To attain all these, care has been taken to establish rapport with every participant prior to interviews. Women and men were interviewed by women and men investigators, respectively. Individual verbal informed consent was obtained from all participants by explaining the purpose of the study. These field works were carried out during September 2004–July 2005.

### Sampling

**Figure 1:**
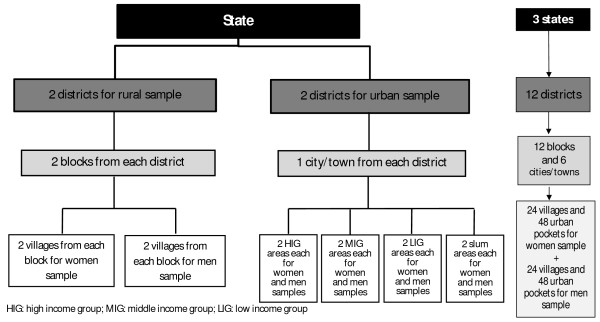
**Multistage sampling adopted for sampling of women and men**.

The sample size was calculated based on the available estimated prevalence of domestic violence for these states [28]. Based on the prevalence of domestic violence, with a confidence level of 95% and absolute precision of 0.05, the samples required were: 450 women for Orissa, 740 women for West Bengal and 480 women for Jharkhand [29]. Same sample sizes were considered for men sample. Keeping in view of 70:30 ratio of rural and urban population, the samples were distributed accordingly. Multistage sampling strategy was used to attain the required samples (Figure 1). From each state, four districts were selected from different corners of the state. Out of these four districts, two each were allocated to draw rural and urban sample. From each district chosen for rural sample, two blocks (administrative units in the district) were selected randomly. From each block, two villages were randomly selected from the list of villages in the block. These two villages were considered for sampling of women participants. In addition, two more villages of similar type and size nearer to the selected village were identified and men were sampled from them. From each district allocated for urban sample, an urban area (a city or a town) was selected. In each urban area, sixteen pockets belonging to different socio-economic strata were identified. These strata were high-income group, middle-income group, low-income groups and slums and were identified based on the information obtained from the local key-informants and physical appearance of housing. Of these 16 pockets, eight (two each from each stratum) pockets each were allotted to sample male and female participants. Thus, from each state, 16 villages and 32 urban pockets were chosen for sampling of female and male participants.

After selecting the village/urban pocket, the research team met village/community heads and elders before initiating the data collection, and the purpose of the survey was explained. Rapport is established with the community and especially the women were taken to the confidence. The sample to be collected from each village was determined by dividing total rural sample required for that state by total number of villages (eight). In each village, eight random points were identified from all corners and care has been taken to include all communities. From each point, required number of sample was collected from households spread in four directions of the point. Similarly, in each urban pocket, participants were selected from the households spread in all the four directions. A married woman up to the age of 50 years of sampled household was sampled from each household. Corresponding to the women sample, married men aged below 50 years were selected in the similar way from the neighbouring village/urban pocket. Initially, 1753 women and 1730 men were contacted; however, 35 women and 15 men have refused to participate, yielding a refusal rate of 2% and 0.8% among women and men, respectively. Thus, samples of 1718 women and 1715 men were obtained.

### Measurements

#### Outcome variables

Three principle domestic violence outcome variables considered in our analysis are: physical violence, psychological violence and sexual violence. They were determined by response to a set of questions for each outcome variable. If a woman (as a victim)/man (as a perpetrator) gave a positive response to any of the questions in a set, it is considered as violence of that category. The questions used for women and men were listed in Annexure 1a and 1b, respectively in Additional file 1. In addition, the fourth variable, i.e. any form of domestic violence was derived. If at least one of the three forms of domestic violence (physical and/or psychological and/or sexual) was present, it was considered as the presence of any form of domestic violence. During logistic regression analyses, these outcome variables were dichotomised into presence and absence of violence, for each type of violence.

#### Socio-economic variables

Data were collected on a number of community-level and individual-level variables that have been linked to domestic violence. The community-level variables included are the state of residence (Orissa, West Bengal or Jharkhand), residence (living in rural or urban), religion (Hindu, Muslim, Christian or any other religion) and caste. During the survey, individual caste of the respondent was collected and they were categorized subsequently during analysis. The Government of India had categorised some ethnic groups (castes and tribes) into scheduled castes, scheduled tribes and backward castes, and these categories are entitled for positive discrimination in educational, employment and other developmental opportunities for their upliftment. The uncategorized castes, which form the majority of the population, are often referred to as forward castes. The individual-level variables were: age in years (which was categorized into individuals less than 20 years of age, those between 20 and 29 years, and those above the age of 30 years), education, which was categorized in to illiterate (those who can neither read nor write), functional literate (those who can read or write, but did not have formal schooling), school education (1–10 years of schooling) and, college education and above (those having more than 10 years of education). The occupation of the participant was recorded and the responses were categorized into salaried jobs (those in permanent or temporary assured jobs with fixed monthly salary), farming and small business (those engaged in agriculture-related activity and small businesses), labourer (daily-waged skilled and unskilled labourers), housewives (only women) and other occupations. The monthly income of the family was calculated during data analysis based on the information collected on income of all members as well as from common sources of the family. The income details were collected in Indian Rupees (INR). One INR was equivalent to 0.02 United States Dollars (US$). For logistic regression, these variables were used as categorical variables, except the age. The categories under each variable were explained above. The age was taken as continuous variable.

### Data Management and Analysis

The data were computerized through Epi-Info 6. The database of Epi-Info was exported to SPSS and further analysis was carried out. The prevalence with 95% confidence intervals (CI) of different forms of domestic violence reported by women and men were computed for each of the states. For the domestic violence prevalence reported by women, the associations with socio-economic variables (habitat, age, religion, caste category, education, occupation and family income) were examined by using both bivariate and multivariate procedures. For each of the group under a variable, the prevalences in the form of percentages were presented and bivariate logistic regressions were carried out. In addition, multiple logistic regression analysis was used to model the presence or absence of physical, psychological and sexual violence, and any form of domestic violence by all of the aforementioned socio-economic variables. For these logistic regression analyses, the dependent variables were dichotomised (presence or absence of violence). The independent variables were categorised into different groups as described under measurements. While calculating odds ratios (OR), the category with the lowest weight was taken as the reference category. The OR is the value by which odds of the event (occurrence of violence) change when the independent variable increases by one unit/step. And it has been calculated by adjusting for all other independent variables in multivariate models. A *p *value of less than 0.05 was considered as the minimum level of significance.

### Ethical considerations

The study protocol has been approved by the Human Ethical Committee of Regional Medical Research Centre. Individual informed consent was obtained from all participants, as mentioned above. Guidelines of World Health Organization, including the importance of ensuring confidentiality and privacy, both as means to protect the safety of study participants and field staff, and to improve the quality of the data were followed [30].

## Results

### Socio-economic characteristics of the participants

The details of socio-economic characteristics of sampled women and men participants were presented in Table 1. Majority of women participants were in the age group of 20–29 years (60%) and men participants were in the age group of 30 years and above (75%). Most of the men and women participants were Hindu. A considerable number of women (6.5%) and men participants (18%) belonged to other than these three major religions. And most of them were from tribal religion, and some were from Sikhism, Jainism and Buddhism. Majority of the participants were from uncategorized castes (forward castes). Regarding educational status, about half of the participants were having school education. Majority of women participants were house-wives. With regard to income, majority participants possessed monthly family income of less than INR 2000 (≅US$ 40).

**Table 1:** Socio-economic characteristics of sampled women and men participants

**Characteristic**	**Female participants** **Number (%)**	**Male participants** **Number (%)**
State		
Orissa	463 (26.9)	466 (27.2)
West Bengal	747 (43.5)	753 (43.9)
Jharkhand	508 (29.6)	496 (28.9)

Residence		
Rural	1200 (69.8)	1200 (70.0)
Urban	518 (30.2)	515 (30.0)

Age group		
< 20 years	126 (7.3)	4 (0.2)
20–29 years	1029 (59.9)	427 (24.9)
30 years and above	563 (32.8)	1284 (74.9)

Religion		
Hindu	1361 (79.2)	1272 (74.2)
Muslim	223 (13.0)	134 (7.8)
Christian	23 (1.3)	4 (0.2)
Others	111 (6.5)	305 (17.8)

Caste category		
Uncategorised castes	817 (47.6)	566 (33.0)
Backward castes	375 (21.8)	430 (25.1)
Scheduled castes	381 (22.2)	345 (20.1)
Scheduled tribes	145 (8.4)	374 (21.8)

Education		
Illiterate	520 (30.3)	451 (26.3)
Functional literate	130 (7.6)	25 (1.4)
School education	818 (47.6)	924 (53.9)
College education and above	250 (14.5)	315 (18.4)

Occupation		
Salaried jobs	61 (3.6)	474 (27.6)
Farming/small business	86 (5.0)	532 (31.0)
Labourer	196 (11.4)	590 (34.4)
Housewife	1375 (80.0)	--
Others	--	119 (6.9)

Family income per month		
< INR 2000	714 (41.6)	729 (42.5)
INR 2001–4000	510 (29.7)	579 (33.0)
INR 4001–6000	140 (8.1)	110 (6.4)
INR 6001–8000	117 (6.8)	73 (4.3)
INR 8001–10000	75 (4.4)	79 (4.6)
> INR 10000	162 (9.4)	145 (8.5)

Total sample	1718	1715

INR: Indian Rupee, approximately equivalent to US$ 0.02

### Prevalence of different forms of domestic violence as reported by women and men

The prevalence of physical, psychological, sexual and any form of domestic violence in the life time of women were presented in Table 2. The life time occurrence of physical violence reported by women was highest in Jharkhand (21.1%), followed by West Bengal (14.6%) and Orissa (13.2%). Psychological violence has been reported by slightly more than half of the women in all the states. Highest prevalence of sexual violence during the life time as reported by women was 32.4% in Orissa, followed by Jharkhand (27.4%) and West Bengal (19.7%). The overall prevalence of physical, psychological, sexual and any form of violence during the life time among Eastern Indian women were 16%, 52%, 25% and 56%, respectively.

**Table 2:** Prevalence of violence (life-time occurrence) as reported by women and men by state

	**Physical violence**	**Psychological violence**	**Sexual violence**	**Any form of violence**
**Reported by women**				

Orissa	13.2 (10.3–16.7)	52.5 (47.8–57.1)	32.4 (28.2–36.9)	60.7 (56.1–65.1)

West Bengal	14.6 (12.2–17.4)	50.6 (47.0–54.2)	19.7 (16.9–22.7)	51.8 (48.2–55.4)

Jharkhand	21.1 (17.6–24.9)	54.5 (50.1–58.9)	27.4 (23.6–31.5)	58.9 (54.4–63.1)

Eastern India	16.1 (14.4–18.0)	52.3 (49.9–54.6)	25.4 (23.3–27.5)	56.3 (53.9–58.6)

**Reported by men**				

Orissa	21.0 (17.5–25.1)	62.7 (58.1–67.0)	17.8 (14.5–21.7)	62.9 (58.3–67.2)

West Bengal	19.4 (16.7–22.4)	53.1 (49.5–56.7)	15.1 (12.7–17.9)	53.1 (49.5–56.7)

Jharkhand	26.4 (22.6–30.6)	65.9 (61.5–70.1)	19.3 (16.0–23.2)	66.1 (61.7–70.2)

Eastern India	21.9 (19.9–23.9)	59.4 (57.0–61.7)	17.1 (15.3–19.0)	59.5 (57.2–61.9)

All figures are percentages; figures in parenthesis are 95% confidence intervals.

Similarly, men were also interviewed to know whether they perpetrated any violence during their life time against their wives (Table 2). The perpetration of physical violence during their life time reported by men was highest in Jharkhand (26.4%), followed by Orissa (21%) and West Bengal (19.4%). Perpetration of psychological violence was also highest in Jharkhand (66%), followed by Orissa (62.7%) and West Bengal (53.1%). The sexual violence, as reported by men as perpetrator during their life time was 19.3% (in Jharkhand), 17.8% (in Orissa) and 15.1% (in West Bengal). Men reported slightly, but not significantly higher prevalence of physical and psychological violence than those reported by women. However, men reported lower prevalences of sexual violence compared to those reported by women.

### Persons responsible for perpetration of domestic violence

It was probed from the women about the person, who actually perpetrated different violent behaviour. Table 3 reveals that husband was mostly responsible for violence among majority of women. Some women reported that in-laws (husbands' parents) were also responsible for few acts of violence, particularly of psychological violence. In Jharkhand, sibs of women's husband were also involved. Few cases of physical violence wherein in-laws and husbands' kins involved were reported from West Bengal and Jharkhand. One woman each from West Bengal and Jharkhand reported to be coerced to sex by their fathers-in-law.

**Table 3:** Involvement of husband in perpetrating different behaviours of domestic violence as reported by women

	**Orissa**		**West Bengal**		**Jharkhand**	
	
**Behaviours of domestic violence**	**Total prevalence**	**By husband**	**Total prevalence**	**By husband**	**Total prevalence**	**By husband**
**Physical violence**						

Hit and beat (including pushed, pulled, slapped, punched, kicked)	13.2	13.0	14.6	14.6	20.9	20.3

Scaled/burnt	0.9	0.9	0.0	0.0	0.2	0.2

**Psychological violence**						

Insulted using abusive language	46.7	41.3	44.3	42.6	50.4	47.0

Threatened with objects like stone, belt, knife, etc.	7.3	7.3	5.6	5.6	8.1	7.5

Threatened to send to parents	14.5	13.0	10.8	10.4	15.4	14.4

Sent to parents' home	1.9	1.3	0.7	0.7	1.0	1.0

Financial hardships	13.8	13.2	17.3	17.1	20.3	19.5

Frightening/angry look	46.9	41.9	48.7	46.9	51.2	48.2

Proved unfaithful	5.2	3.9	4.6	4.3	4.9	4.5

Showed indifference	10.6	8.9	18.6	17.1	20.1	19.5

Deprived of privileges	5.2	4.5	4.8	4.7	8.3	7.7

Neglected	23.3	20.3	22.5	21.3	28.9	28.0

Denial of basic personal needs	6.9	6.7	6.4	6.3	9.3	9.1

Non involvement in decision making	28.5	24.8	15.3	18.9	36.0	34.4

Restriction in mobility	4.8	4.1	1.9	1.6	6.9	6.3

**Sexual violence**						

Coerced sex	30.9	30.7	19.0	19.0	26.2	26.0

Denial of sex	5.2	5.2	0.8	0.8	1.6	1.6

Causing sexual hurt/injury	1.1	1.1	0.8	0.8	1.4	1.4

### Continuation of domestic violence

It was probed to know whether or not the reported behaviours of violence are continuing currently among the women, who reported the experience of different acts of physical, psychological and sexual violence during their lifetime. If it is continuing, it was further probed for each act to know the periodicity of their occurrence. It is probed to know whether they are experiencing these acts daily. It is found that, almost all acts of violence were still continuing among majority of women (Table 4). For example, the insult of women through abusive language is reported to be continuing among 41.3% of women of Orissa, where as 23.8% of women reported that they were experiencing daily. Similar situation was reported for all behaviours of violence, including sexual coercion which is continuing among 27% out of 31% of women of Orissa, 16% out of 19% of women of West Bengal and 22% out of 26% of women of Jharkhand. A majority of women reported that they were experiencing these acts of violence daily.

**Table 4:** Continuation and frequency of different behaviours of domestic violence as reported by women

	**Orissa**			**West Bengal**			**Jharkhand**		
	
**Behaviours of domestic violence**	**Total prevalence**	**Continuing**	**Daily**	**Total prevalence**	**Continuing**	**Daily**	**Total prevalence**	**Continuing**	**Daily**
**Physical violence**									

Hit and beat (including pushed, pulled, slapped, punched, kicked)	13.2	12.3	2.8	14.6	13.1	2.4	20.9	17.5	4.1

Scaled/burnt	0.9	0.6	0.4	0.0	0.0	0.0	0.2	0.0	0.0

**Psychological violence**									

Insulted using abusive language	46.7	41.3	23.8	44.3	41.8	22.8	50.4	45.3	32.3

Threatened with objects like stone, belt, knife, etc.	7.3	6.9	2.6	5.6	5.2	0.4	8.1	7.1	2.8

Threatened to send to parents	14.5	12.1	4.5	10.8	9.1	2.9	15.4	12.6	6.3

Sent to parents' home	1.9	1.7	0.9	0.7	0.7	0.0	1.0	0.4	0.4

Financial hardships	13.8	12.7	10.8	17.3	16.2	13.3	20.3	18.1	15.6

Frightening/angry look	46.9	41.9	27.2	48.7	46.9	29.5	51.2	41.2	35.4

Proved unfaithful	5.2	5.0	3.9	4.6	3.6	1.3	4.9	3.7	2.4

Showed indifference	10.6	8.6	5.6	18.6	17.0	6.6	20.1	17.5	8.5

Deprived of privileges	5.2	4.8	3.5	4.8	4.1	0.5	8.3	7.1	3.0

Neglected	23.3	15.1	14.5	22.5	0.0	0.0	28.9	0.0	10.8

Denial of basic personal needs	6.9	6.7	4.5	6.4	5.9	2.4	9.3	8.1	4.5

Non involvement in decision making	28.5	26.1	21.0	15.3	19.1	15.1	36.0	33.9	29.5

Restriction in mobility	4.8	4.8	3.0	1.9	1.5	0.8	6.9	5.9	3.0

**Sexual violence**									

Coerced sex	30.9	27.0	11.7	19.0	15.9	6.8	26.2	21.7	7.4

Denial of sex	5.2	4.5	0.9	0.8	0.5	0.5	1.6	0.8	1.0

Causing sexual hurt/injury	1.1	0.9	0.2	0.8	0.7	0.0	1.4	1.4	0.2

### Prevalence of domestic violence by socio-economic characteristics of women

Table 5 illustrates the prevalence of various forms of domestic violence during the life time reported by women by different socio-economic characteristics. In each category, percentage of women experienced violence to the total number of women belonged to that particular category of socio-economic characteristic was given. The rural-urban differences were slightly visible. Urban women reported slightly higher prevalences of physical and psychological violence as well as overall domestic violence. However, the prevalence of sexual violence was slightly higher among rural women. Age has a profound association with the prevalence of domestic violence in these communities. Prevalences of all forms of violence were increased along with the age of the women. Women aged 20–29 years and aged above 29 years have reported higher prevalence of violence than women aged less than 20 years. The differences among various religious groups were not conspicuous. However, there were apparent differences across the groups categorised based on their caste/tribe affiliation. Women belonged to backward castes reported higher prevalence of any type of violence along with psychological and sexual violence. However, scheduled tribes also reported higher prevalences of all sorts of violence. The data revealed that education has impact on the prevalence of domestic violence. The prevalence of violence decreased as educational levels of women increased. Also, there were variations in the prevalence of violence across different occupational groups of women. Higher prevalence of violence was reported by women who were engaged in farming and small business. Women with lowest income reported highest prevalence of violence. However, the prevalences were higher among high-income groups than among middle-income groups.

**Table 5:** Prevalence of various forms of domestic violence reported by women by some socio-economic characteristics

	Prevalence of domestic violence*			
	
**Characteristics**	**Physical violence**	**Psychological violence**	**Sexual violence**	**Any form of violence**
Residence				
Rural	15.7	51.3	25.6	55.7
Urban	17.2	54.4	24.9	57.7

Age group				
< 20 years	2.38	29.4	7.9	30.2
20–29 years	14.4	51.4	22.8	53.9
30 years and above	22.4	59.0	33.9	66.4

Religion				
Hindu	15.4	51.9	25.1	56.4
Muslim	17.0	53.4	25.1	55.6
Christian	8.7	47.8	17.4	47.8
Others	25.2	55.9	31.5	58.6

Caste category				
General castes	12.7	48.2	22.0	51.5
Backward castes	21.6	59.5	30.4	64.8
Scheduled castes	14.4	53.3	26.0	58.5
Scheduled tribes	25.5	53.8	29.7	55.2

Education				
Illiterate	26.2	59.0	32.7	62.7
Functional literate	13.1	53.8	30.8	60.8
School education	13.6	51.5	22.5	55.6
College education and above	5.2	40.0	16.8	42.8

Occupation				
Salaried job	3.3	52.5	13.1	52.5
Farming/small business	34.9	75.6	25.6	75.6
Labourer	22.4	56.1	27.0	56.6
Housewife	14.6	50.3	25.7	55.2

Family income per month				
< INR 2000	20.3	57.7	27.9	60.9
INR 2001–4000	17.8	50.4	27.1	56.7
INR 4001–6000	8.6	45.7	17.9	50.0
INR 6001–8000	10.3	39.3	22.2	43.6
INR 8001–10000	9.3	48.0	21.3	54.7
> INR 10000	6.2	51.2	19.8	54.9

Total	16.1	52.3	25.4	56.3

INR: Indian Rupee, approximately equivalent to US$ 0.02

*Prevalence is percentage of women reported violence to the total number of women in that category of socio-economic characteristic.

The above associations were further examined through bivariate logistic regressions by taking presence or absence of violence as a dependent variable and women's socio-economic characteristic as a covariate (independent variables). OR along with levels of significance of regression models for all types of violence are shown in Table 6. A significant association was found between presence of physical violence and women's characteristics namely, state, age, religion, caste, education and monthly family income. The psychological and sexual violence also showed significant association with these variables except with state and religion. Psychological violence yielded significant regression coefficient with women's occupation. The variable, any form of violence recorded significant regression coefficients with age, caste, education and monthly family income. The OR obtained for association of violence occurrence with education and income are below one and they revealed that the prevalence of violence decreases along with the increase of women's education and family income.

**Table 6:** Results of bivariate logistic regression between socio-economic characteristics and prevalence of various forms of domestic violence reported by women

	OR* (95% CI)			
	
**Characteristic**	**Physical violence**	**Psychologi-cal violence**	**Sexual violence**	**Any form of violence**
State (*Orissa = 1, West Bengal = 2, Jharkhand = 3*)	1.35*** (1.13–1.60)	1.04 (0.92–1.18)	0.88 (077–1.02)	0.97 (0.88–1.10)

Residence (*rural = 1, urban = 2*)	1.12 (0.85–1.47)	1.13 (0.92–1.39)	0.96 (0.76–1.22)	1.09 (0.88–1.34)

Age (*in years*)	1.10*** (1.08–1.13)	1.07*** (1.05–1.09)	1.10*** (1.07–1.12)	1.09*** (1.07–1.11)

Religion (*Hindu = 1, Muslim = 2, Christian = 3, others = 4*)	1.19* (1.03–1.38)	1.05 (0.93–1.18)	1.08 (0.94–1.23)	1.01 (0.89–1.14)

Caste (*uncategorised caste = 1, backward caste = 2, scheduled caste = 3, scheduled tribe = 4*)	1.22*** (1.08–1.38)	1.10* (1.00–1.21)	1.13* (1.01–1.26)	1.11* (1.01–1.22)

Education level (*illiterate = 1, functional literate = 2, school education = 3, college education and above = 4*)	0.62*** (0.55–0.70)	0.81*** (0.74–0.88)	0.76*** (0.68–0.84)	0.80*** (0.73–0.88)

Occupation (*salaried job = 1, farming/small business = 2, labourer = 3, housewife = 4*)	1.17 (0.99–1.38)	1.24*** (1.08–1.42)	0.89 (0.76–1.04)	1.13 (0.99–1.29)

Monthly family income (*<INR 2000 = 1; INR 2001–4000 = 2; INR 4001–6000 = 3; INR 6001–8000 = 4; INR 8001–10000 = 5; >INR 10000 = 6*)	0.76*** (0.68–0.84)	0.92*** (0.86–0.97)	0.90*** (0.84–0.97)	0.93** (0.87–0.98)

OR: odds ratio; CI: confidence interval

*The category with the lowest weight is the reference category for calculating OR. And OR is the value by which odds of the event change when the independent variable increases by one unit/step.

Significance of regression model: *p < 0.05, **p < 0.01, ***p < 0.001

Further, multivariate logistic regressions were carried out to examine these associations, separately for each type of domestic violence (Tables 7, 8, 9 and 10). The physical violence has significant association with state, residence (rural or urban), age and occupation of women, and monthly family income (Table 7). The association between occurrence of physical violence and the family income was inverse, as occurrence of violence decreased with increasing family income. Psychological violence was significantly associated with residence, age, education and occupation of the women and monthly family income (Table 8). However, only residence, age and educational level of women were significantly associated with the occurrence of sexual violence (Table 9). Regression analysis for occurrence of any form of violence revealed that residence, age, educational level and occupation of women and monthly family income were significantly associated (Table 10).

**Table 7:** Details of logistic regression to examine the association of socio-economic variables of women on the prevalence of physical violence reported by women

**Socio-economic variable**	**Coefficient ± SE**	Adjusted OR* (95% CI)
Constant	-6.81 ± 0.82***	--

State (*Orissa = 1, West Bengal = 2, Jharkhand = 3*)	0.31 ± 0.10***	1.36 (1.13–1.64)

Residence (*rural = 1, urban = 2*)	1.12 ± 0.19***	3.06 (2.11–4.44)

Age (*in years*)	0.11 ± 0.01***	1.12 (1.09–1.15)

Religion (*Hindu = 1, Muslim = 2, Christian = 3, others = 4*)	0.16 ± 0.08	1.17 (0.99–1.39)

Caste (*uncategorised caste = 1, backward caste = 2, scheduled caste = 3, scheduled tribe = 4*)	0.06 ± 0.08	1.06 (0.91–1.24)

Education level (*illiterate = 1, functional literate = 2, school education = 3, college education and above = 4*)	-0.360 ± 0.08***	0.70 (0.59–0.82)

Occupation (*salaried job = 1, farming/small business = 2, labourer = 3, housewife = 4*)	0.25 ± 0.10**	1.28 (1.05–1.56)

Monthly family income (*<INR 2000 = 1; INR 2001–4000 = 2; INR 4001–6000 = 3; INR 6001–8000 = 4; INR 8001–10000 = 5; >INR 10000 = 6*)	-0.37 ± 0.07***	0.69 (0.61–0.79)

SE: standard error; OR: odds ratio; CI: confidence interval, INR: Indian Rupee, approximately equivalent to US$ 0.02

Significance of regressions coefficients: *p < 0.05, **p < 0.01, ***p < 0.001

*The category with the lowest weight is the reference category for calculating OR. And OR is the value by which odds of the event change when the independent variable increases by one unit/step, adjusting for all other independent variables in the multivariate model.

**Table 8:** Details of logistic regression to examine the association of socio-economic variables of women on the prevalence of psychological violence reported by women

**Socio-economic variable**	**Coefficient ± SE**	Adjusted OR* (95% CI)
Constant	-2.21 ± 0.55***	--

State (*Orissa = 1, West Bengal = 2, Jharkhand = 3*)	0.03 ± 0.07	1.03 (0.90–1.17)

Residence (*rural = 1, urban = 2*)	0.55 ± 0.14***	1.74 (1.32–2.30)

Age (*in years*)	0.07 ± 0.01***	1.07 (1.04–1.09)

Religion (*Hindu = 1, Muslim = 2, Christian = 3, others = 4*)	0.03 ± 0.07	1.03 (0.90–1.17)

Caste (*uncategorised caste = 1, backward caste = 2, scheduled caste = 3, scheduled tribe = 4*)	0.04 ± 0.06	1.04 (0.93–1.17)

Education level (*illiterate = 1, functional literate = 2, school education = 3, college education and above = 4*)	-0.19 ± 0.06**	0.83 (0.73–0.93)

Occupation (*salaried job = 1, farming/small business = 2, labourer = 3, housewife = 4*)	0.26 ± 0.07***	1.30 (1.12–1.49)

Monthly family income (*<INR 2000 = 1; INR 2001–4000 = 2; INR 4001–6000 = 3; INR 6001–8000 = 4; INR 8001–10000 = 5; >INR 10000 = 6*)	-0.15 ± 0.04***	0.86 (0.79–0.94)

SE: standard error; OR: odds ratio; CI: confidence interval, INR: Indian Rupee, approximately equivalent to US$ 0.02

Significance of regressions coefficients: *p < 0.05, **p < 0.01, ***p < 0.001

*The category with the lowest weight is the reference category for calculating OR. And OR is the value by which odds of the event change when the independent variable increases by one unit/step, adjusting for all other independent variables in the multivariate model.

**Table 9:** Details of logistic regression to examine the association of socio-economic variables of women on the prevalence of sexual violence reported by women

**Socio-economic variable**	**Coefficient ± SE**	Adjusted OR* (95% CI)
Constant	-2.90 ± 0.63***	--

State (*Orissa = 1, West Bengal = 2, Jharkhand = 3*)	-0.12 ± 0.08	0.88 (0.75–1.03)

Residence (*rural = 1, urban = 2*)	0.34 ± 0.16*	1.41 (1.03–1.92)

Age (*in years*)	0.10 ± 0.01***	1.10 (1.07–1.12)

Religion (*Hindu = 1, Muslim = 2, Christian = 3, others = 4*)	0.09 ± 0.07	1.09 (0.94–1.27)

Caste (*uncategorised caste = 1, backward caste = 2, scheduled caste = 3, scheduled tribe = 4*)	0.10 ± 0.07	1.11 (0.97–1.27)

Education level (*illiterate = 1, functional literate = 2, school education = 3, college education and above = 4*)	-0.24 ± 0.07***	0.79 (0.68–0.90)

Occupation (*salaried job = 1, farming/small business = 2, labourer = 3, housewife = 4*)	-0.13 ± 0.09	0.87 (0.73–1.04)

Monthly family income (*<INR 2000 = 1; INR 2001–4000 = 2; INR 4001–6000 = 3; INR 6001–8000 = 4; INR 8001–10000 = 5; >INR 10000 = 6*)	-0.07 ± 0.05	0.93 (0.84–1.03)

SE: standard error; OR: odds ratio; CI: confidence interval, INR: Indian Rupee, approximately equivalent to US$ 0.02

Significance of regressions coefficients: *p < 0.05, **p < 0.01, ***p < 0.001

*The category with the lowest weight is the reference category for calculating OR. And OR is the value by which odds of the event change when the independent variable increases by one unit/step, adjusting for all other independent variables in the multivariate model.

**Table 10:** Details of logistic regression to examine the association of socio-economic variables of women on the prevalence of any form of domestic violence reported by women

**Socio-economic variable**	**Coefficient ± SE**	Adjusted OR* (95% CI)
Constant	-1.46 ± 0.65*	--

State (*Orissa = 1, West Bengal = 2, Jharkhand = 3*)	-0.03 ± 0.07	0.97 (0.84–1.11)

Residence (*rural = 1, urban = 2*)	0.45 ± 0.14***	1.56 (1.18–2.07)

Age (*in years*)	0.09 ± 0.01***	1.10 (1.07–1.12)

Religion (*Hindu = 1, Muslim = 2, Christian = 3, others = 4*)	-0.01 ± 0.07	0.99 (0.87–1.14)

Caste (*uncategorised caste = 1, backward caste = 2, scheduled caste = 3, scheduled tribe = 4*)	0.09 ± 0.06	1.10 (0.97–1.24)

Education level (*illiterate = 1, functional literate = 2, school education = 3, college education and above = 4*)	-0.20 ± 0.06***	0.82 (0.73–0.93)

Occupation (*salaried job = 1, farming/small business = 2, labourer = 3, housewife = 4*)	-0.15 ± 0.07*	0.86 (0.75–0.99)

Monthly family income (*<INR 2000 = 1; INR 2001–4000 = 2; INR 4001–6000 = 3; INR 6001–8000 = 4; INR 8001–10000 = 5; >INR 10000 = 6*)	-0.11 ± 0.04**	0.89 (0.82–0.97)

SE: standard error; OR: odds ratio; CI: confidence interval, INR: Indian Rupee, approximately equivalent to US$ 0.02

Significance of regressions coefficients: *p < 0.05, **p < 0.01, ***p < 0.001

*The category with the lowest weight is the reference category for calculating OR. And OR is the value by which odds of the event change when the independent variable increases by one unit/step, adjusting for all other independent variables in the multivariate model.

## Discussion

In the present study, women reported as high as 56% of some form of violence against them in Eastern part of India. The levels of physical, psychological and sexual violence against women were also considerably high. These data along with the world-wide literature confirm that domestic violence is a universal phenomenon existing in all communities [8,12,31]. Also, it is confirmed that women were at more risk of violence by their husband than any other perpetrator. However, these figures should be understood cautiously as some of the behaviours considered as violent behaviour (such as coerced sex by husband-husband having sex with his wife when she is unwilling) may not be perceived by either partners or people as being inappropriate or wrongful [32]. However, irrespective of the people's perceptions, these behaviours have influence on both physical and mental health of women.

The present data demonstrated that in Eastern India, the domestic violence is persisting considerably across all socio-economic strata. Some characteristics of women namely, residence, age, education, occupation and family income have influence on the prevalence of domestic violence. The prevalence of violence decreased along with the increase of women's education and family income. However, no comprehensive studies are available from this part of India to compare these findings. One nation-wide study from India revealed that higher socio-economic status as a protective buffer against domestic violence [16]. The data from Uttar Pradesh, a north Indian state revealed similar results on association of domestic violence with socio-economic characteristics [12,33]. But these data were collected from the perspective of men. These studies revealed that higher levels of education among both husbands and wives and greater household wealth were found to be protective factors against the risk of physical violence. But no such association was evident with respect to sexual violence, and in fact women married to more educated men experienced significantly higher risk of coercive sex [34].

Some of the earlier studies from India revealed that though inadequate and failure of timely payment of dowry has been focused as an important reason for domestic violence in India, several other triggers of domestic violence such as negligence or failure in performing duties expected of women in the family also led to violence against women [10]. These causes reflect deep-rooted gender inequalities that persist across India. It is due to male patriarchy, which is defined as a system of male dominance legitimated by within the family and the society through superior rights, privileges, authority and power [35]. Socialisation of women into subordinate position and thinking of men that they are superior to women and have a right to control women are resultant phenomena of male patriarchy. Such socialisation leads to powerlessness of women, which ultimately leads to violence and inability of women to defend themselves [10]. Heise argued that violence is an extension of a continuum of beliefs that grants men the right to control women's behaviour [36]. Miller also suggested that low self-esteem among Indian girls contribute to the women's acceptance of violence by their husbands [37]. In addition, studies conducted during last ten years identified several community and individual level variables that determine the risk of domestic violence [34,38]. In the present study, urban women reported a higher prevalence of violence than rural women. As expected, living in urban areas is a higher risk factor than living in rural areas and as such, the current data corroborate results from other developing nations [39,40]. However, these findings do not confirm with the pattern in India [18]. Urban social environmental conditions can be more stressful, alienating, and anomic than do rural areas and such conditions may influence spousal relations [40]. In Indian communities, higher levels of income and education were found to be protective [16,41-45].

This study, along with the domestic violence rates based on the reporting of women, presented the prevalence of domestic violence reported by men, as perpetrator. These rates are in corroboration with those reported by women. Almost all research on domestic violence has relied on women's rather than men's report of their experiences [32]. Few studies have asked both partners of a couple about their experiences of domestic violence, and they yielded various degrees of consensus [46,47]. However, in the present study, the rates of physical and psychological violence reported by men were more than those reported by women, where as the rates of sexual violence were less than those reported by women. It may be due to the differences in the perceptions of men and women regarding certain behaviours as sexual violence. For example, husband may not perceive coercion as against the will of wife. In the present socio-cultural context, the initiator for sex is usually the husband. To larger extent, sex remained as a hidden subject of discussion even between wife and husband; and women are not expected to express their desire. This prevailing societal norm might have led men to think sex as prerogative of husband and wife is just expected to accept. Probably, men might not have perceived the sexual violence as perceived by women. Heise et al. felt that the meaning of such behaviour may not be perceived by either partner as being inappropriate or wrongful [32]. However, it is not out of context to note that forced sex within the marriage is considered as rape or sexual assault in many countries including several states in the United States. Recently, India, through the Protection of Women from Domestic Violence Act of 2005, recognised different forms of physical, sexual, verbal, emotional or economic abuse as domestic violence. Under this act, rape within the marriage is considered as a crime [48]. Previously it was impossible to prosecute a man for sexually raping his wife, which was considered to be within his conjugal rights. High level of normative support and limited/absence of community sanctions on violence against wife in these communities might have made men to report, and also these rates were comparable with those reported by women. A similar agreement between partners in reporting of physical violence was reported by other studies [49,50]. Hence, investigating men may be used as an element of validation of estimates of domestic violence. Also, it may be relied on the reporting of men in communities, where investigating women is difficult.

### Methodological considerations

There are limitations in this study, as usual to this type of research topic. The topic of interview is very sensitive and participants may not express their views openly, as they think that their responses may damage the reputation of themselves and their families. Sometimes in this type of research, participants may also report the behaviour that is believed to be consistent with their culture, rather than the actual [51]. However, these were managed by the trained field staff by interviewing the participants alone. Like any study based on the self-reporting, there might be recall bias in disclosing the violent episodes. Since Indian women are usually stigmatized and blamed for the violence and abuse they receive, as well as for their husbands' violent behaviour, over-reporting of violence is unlikely. However, there is possibility of risk of potential bias as respondents' willingness to disclose their violence experiences vary across different socio-economic groups. Another limitation is the cross-sectional design itself, which do not allow for making conclusions focused on associations. It is difficult to make causal inferences. However, the direction of some of the associations like association of violence with women's caste and religion are expected. The associations between occurrence of violence and family income and women's occupation might be a 'both ways' association. Despite these limitations, the study had methodological strengths including use of standardized pre-tested instruments, inclusion of all groups of population, rigorous training to field workers and establishment of rapport with the study communities and participants.

## Conclusion

The study confirms the high prevalence of all forms of violence against women across all socio-economic settings in eastern zone of India. However, urban residence, older age, lower education and lower family income are associated with occurrence of domestic violence. Women are at risk of violence from the husband than any other type of perpetrator. This situation has public health implications as public health can have a role in preventing the violence and its health consequences. Also, the primary healthcare institutions in India should institutionalise the routine screening and treatment for violence related injuries and trauma. These results also provide vital information to assess the situation to develop interventions as well as policies and programmes towards preventing violence against women. As India has already passed a bill against domestic violence, the present results on robustness of the problem will be useful to sensitise the concerned agencies to strictly implement the law.

## Competing interests

The authors declare that they have no competing interests.

## Authors' contributions

Both the authors contributed to the conception of the study design and development of study instruments. BVB involved in field works; coordinated in the data collection; computerized and analysed the data; interpreted the results; prepared the manuscript. Both the authors read and approved the final manuscript.

## Pre-publication history

The pre-publication history for this paper can be accessed here:



## Supplementary Material

Additional file 1 **Annexure 1**. A: Questions posed to women in this study to consider physical, psychological and sexual violence against women. B: Questions posed to men in this study to consider physical, psychological and sexual violence against their wives Click here for file
