# Supplementary material for: Domestic violence against women in eastern India: a population-based study on prevalence and related issues
Source: BMC Public Health. 2009 May 9;9:129. doi: 10.1186/1471-2458-9-129 (PMC2685379; doi:10.1186/1471-2458-9-129)
Supplement: Additional file 1 — Annexure 1. A: Questions posed to women in this study to consider physical, psychological and sexual violence against women. B: Questions posed to men in this study to consider physical, psychological and sexual violence against their wives [file 1471-2458-9-129-S1.doc]

**Annexure 1a: Questions posed to women in this study to consider physical, psychological and sexual violence against women**

| **Psychological violence** |
| --- |
| 1. Has your husband or other family members ever insulted you by using abusive language? 2. Has your husband or other family members ever threatened you with objects like stick, belt, knife, etc.? 3. Has your husband or other family members ever threatened you to send to your parents’ house? 4. Has your husband or other family members ever sent you to your parents’ house? 5. Has your husband or other family members ever created financial hardships to you, by not making money available to you? 6. Has your husband or other family members ever frightened you like looking angrily at you? 7. Is your husband or other family members often suspicious that you are unfaithful? 8. Has your husband or other family members ever ignored you and showed indifference to you? 9. Has your husband or any other family member ever made you deprived of your privileges in the family? 10. Has your husband or any other family member ever neglected you? 11. Have you ever been denied of basic personal needs? 12. Have you ever felt that you are not being involved in decision makings in the family? 13. Has your husband or any other family member ever restricted you to go to your parents’ home or other places like friends’/relatives’ houses, places of worship, etc.? |
| **Physical violence** |
| 1. Has your husband or any other family member ever assaulted you physically (like slapped or thrown something, pushed, pulled your hair, shoved, hit with fist or something else, kicked, dragged or beaten you up, chocked)? 2. Has your husband or any other family member ever scalded you or burnt you on purpose? |
| **Sexual violence** |
| 1. Did your husband or any other male members in your family ever physically force you to have sexual intercourse when you did not want to? 2. Did your husband wilfully deny or avoid you to have sex? 3. Did your husband ever hurt or cause injury to your private parts? |

**Annexure 1b: Questions posed to men in this study to consider physical, psychological and sexual violence against their wives**

| **Psychological violence** |
| --- |
| 1. Have you ever insulted your wife by using abusive language? 2. Have you ever threatened your wife with objects like stick, belt, knife, etc.? 3. Have you ever threatened your wife to send to her parents’ house? 4. Have you ever sent your wife to her parents’ house? 5. Have you ever created financial hardships to your wife, by not making money available to her? 6. Have you ever frightened your wife like by looking angry at her? 7. Have you ever expressed suspicion to your wife that she is unfaithful to you? 8. Have you ever ignored your wife or shown indifference to your wife? 9. Have you ever made your wife deprived of her privileges in the family? 10. Have you ever neglected your wife? 11. Have you ever denied the basic personal needs to your wife? 12. Have you ever wilfully not involved your wife in decision making in the family? 13. Have you ever restricted your wife to go to her parents’ home or other places like friends’/relatives’ houses, places of worship, etc.? |
| **Physical violence** |
| 1. Have you ever assaulted your wife physically (like slapped or thrown something, pushed, pulled your hair, shoved, hit with fist or something else, kicked, dragged or beaten you up, chocked)? 2. Have you ever scalded your wife or burnt your wife, purposefully? |
| **Sexual violence** |
| 1. Did you ever physically force your wife to have sex when she did not want? 2. Did you ever wilfully deny or avoid sex with your wife? 3. Did you ever hurt or cause injury on private parts of your wife? |
